# Prolonged Adrenal Insufficiency After Failed Cryoablation and Osilodrostat for Cushing Syndrome in Nodular Adrenal Disease

**DOI:** 10.1210/jcemcr/luaf091

**Published:** 2025-04-29

**Authors:** Colleen Veloski, Amanda Sturgeon, Julie Hallanger Johnson

**Affiliations:** Department of Head and Neck—Endocrine Oncology, H Lee Moffitt Cancer Center and Research Institute, Tampa, FL 33612, USA; Department of Head and Neck—Endocrine Oncology, H Lee Moffitt Cancer Center and Research Institute, Tampa, FL 33612, USA; Division of Endocrinology, Diabetes, Metabolism, & Nutrition, Mayo Clinic, Rochester, MN 55905, USA

**Keywords:** Cushing syndrome, adrenal nodule, adrenal adenoma, osilodrostat, adrenal insufficiency

## Abstract

Nodular adrenocortical disease is an entity more commonly recognized in recent years. We present a case of bilateral adrenal nodular disease in a young woman with ACTH-independent Cushing syndrome. She was treated with medical therapies at her preference to avoid adrenal insufficiency (AI) from surgery. She developed intolerance to medical therapy. Cryoablation of the right adrenal nodule was performed after adrenal vein sampling identified the right adrenal as the dominant source of cortisol. Cortisol levels were normal while on medical therapy after cryoablation but quickly became elevated after discontinuing medical therapy. The patient was then treated with osilodrostat and ultimately developed medication-induced AI that has persisted for more than 3 years. Due to the increased availability of new medications to treat Cushing syndrome, we present our experience to educate endocrinology audiences about the unexpected responses to medications. Using osilodrostat (off-label) in this patient led to prolonged primary AI after 4 months of use and now presumed permanent AI 36 months after discontinuation of treatment.

## Introduction

With the explosion in medical management options for Cushing syndrome, particularly with more US Food and Drug Administration-approved medical management options for Cushing disease, this case is presented to highlight some important side effects of new medical management options. The possibility of medication-induced adrenal insufficiency is emphasized to allow early recognition of this life-threatening complication.

## Case Presentation

This 38-year-old woman presented to endocrinology in 2015 with classic symptoms and signs of Cushing syndrome with hypertension, weight gain, truncal obesity, abdominal striae, easy bruising, proximal muscle weakness, hirsutism, acne, and abnormal menstrual cycles.

## Diagnostic Assessment

**Figure 1.**
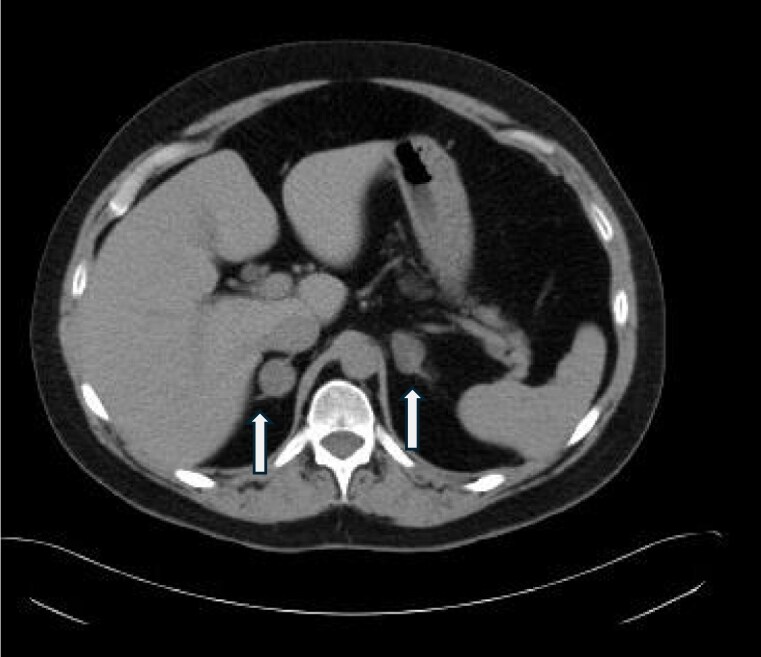
Initial noncontrast computed tomography imaging of the patient from February 18, 2015, shows that the right adrenal gland nodule measured 1.9 × 2.0 cm, 30 HU; left adrenal gland nodule measured 2.4 × 1.6 cm, 15 HU. These were consistent with indeterminate bilateral adrenal nodules. White arrows show the right and left adrenal glands. Abbreviation: HU, Hounsfield units.

Her examination was highly suspicious for Cushing syndrome. Serum ACTH was completely suppressed at < 5 pg/mL; < 1.1 pmol/L (normal range 6-50 pg/mL; 1.3-11 pmol/L), with cortisol of 17.8 µg/dL; 491 nmol/L (early Am sample normal range 4.0-22 µg/dL; 110-607 nmol/L). Cortisol was 18.2 µg/dL, 502 nmol/L (reference range less than 1.8 µg/dL; 50 nmol/L) and a dexamethasone level 559 ng/mL; 14.2 nmol/L (reference range 140-295 ng/dL; 3.6-7.5 nmol/L) after a 1 mg dexamethasone suppression test. Two 24-hour urine free cortisol (UFC) tests were elevated at 159 µg/24 hours; 438.8 nmol/24 hours and 217.9 µg/24 hours; 601 nmol/24 hours (normal range 4-50 µg/24 hours; 11-138 nmol/24 hours). The low ACTH prompted adrenal imaging showing bilateral adrenal nodules with indeterminate features on noncontrast imaging, measuring 2.5 cm on the left and 2.0 cm on the right (Fig. 1).

## Treatment

Surgery was recommended, but the patient decided against adrenal surgery of any type. She was therefore treated with mifepristone, glucocorticoid receptor antagonist therapy, from May 2015 to November 2018 with good control of symptoms. While on treatment with mifepristone, her 24-hour UFC normalized to 7.6 µg/24 hours; 21 nmol/24 hours (normal range 4-50 µg/24 hours; 11-138 nmol/24 hours) by September 2015. This was an unexpected finding given that mifepristone typically results in increased urinary cortisol excretion due to its glucocorticoid receptor antagonistic mechanism of action. Urinary cortisol was intermittently normal and mildly elevated over time. By April 2017, her ACTH had normalized. Unfortunately, she developed endometrial intraepithelial neoplasia, which required discontinuation of the mifepristone in November 2017.

She was enrolled in a phase II clinical trial with a novel glucocorticoid receptor antagonist, CORT 125134, now called relacorilant. She experienced weight gain and hypertension causing her to discontinue treatment. Outside laboratory results during this time demonstrated elevation of the UFC to 5 times the upper normal range. Ultimately, she underwent elective hysterectomy in July 2018, allowing her to resume treatment with mifepristone.

Dose escalation to maximal doses of mifepristone led to laboratory value improvements. By February 2020, she had developed mild hypoglycemia, and her ACTH was 35 pg/mL; 7.7 pmol/L (normal range 6-50 pg/mL; 1.3-11 pmol/L), prompting a dose reduction of mifepristone. UFC remained elevated at 101.8 µg/24 hours; 278.6 nmol/24 hours (normal range 4-50 µg/24 hours; 11-138 nmol/24 hours) as expected given the mechanism of action of mifepristone.

**Figure 2.**
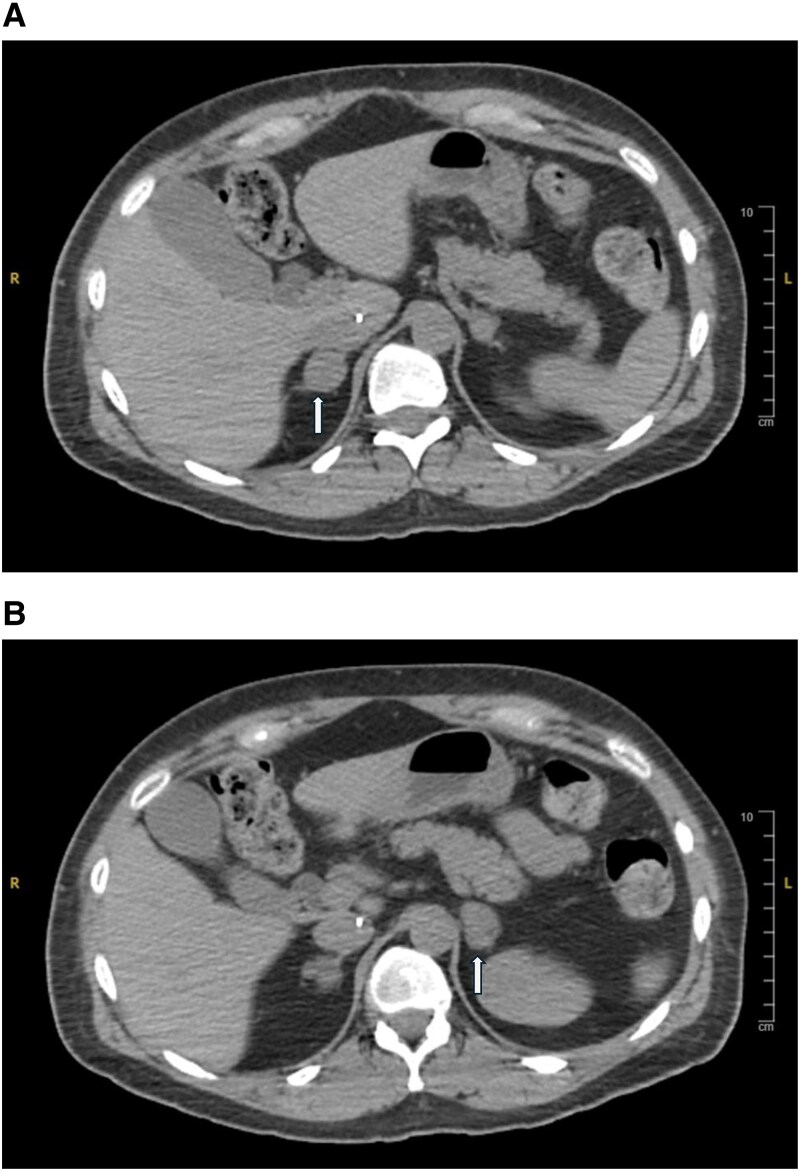
Contrast-enhanced computed tomography of imaging of the patient from October 7, 2020, before adrenal cryoablation without significant change from 2015 imaging. (A) Imaging of right adrenal gland; (B) imaging of left adrenal gland.

Despite improvement in her laboratory studies, she continued to feel unwell and searched for treatment options other than surgery. In consideration of possible unilateral adrenalectomy vs cryoablation, she underwent adrenal venous sampling after 2-day low-dose dexamethasone suppression. Concentrations of cortisol and epinephrine were measured in blood obtained from both adrenal veins and the external iliac vein as outlined in the study by Young et al [1, 2, 3]. The right adrenal was the dominant source (Table 1). Prior to cryotherapy, the right adrenal gland measured 1.7 cm (Fig. 2A), and the left adrenal gland measured 2.4 cm (Fig. 2B). The patient continued to decline surgical treatment and genetic evaluation for her adrenal nodular hyperplasia. She elected to proceed with cryoablation of the right adrenal gland on October 28, 2020, after which she experienced 2 to 3 months with no symptoms and normal ACTH while continuing mifepristone until 24-hour UFC normalized to 10 µg/24 hours; 27.6 nmol/24 hours (normal range 4-50 µg/24 hours; 11-138 nmol/24 hours). Mifepristone was discontinued on December 4, 2020. UFC on March 11, 2021, remained normal at 13 µg/24 hours; 35.9 nmol/24 hours (normal range 4-50 µg/24 hours; 11-138 nmol/24 hours). The response was short-lived, unfortunately.

**Table 1.** Adrenal Venous Sampling Results

Vein	Epinephrine	Cortisol	Cortisol AV:PV ratio	Cortisol lateralization ratio
Right AV	16 967 ng/dL (926 228 pmol/L)	275.8 µg/dL (7609 nmol/L)	16	2.88
Left AV	185 ng/dL (10,099 pmol/L)	95.6 µg/dL (2638 nmol/L)	5.8	
PV (external iliac)	22, 21 ng/dL (1200, 1146 pmol/L)	17.0, 16.4 µg/dL (469, 453 nmol/L)		

Adrenal venous sampling: performed after 2-day low-dose dexamethasone suppression testing.

Confirmation of placement within the AV was considered successful if the AV epinephrine levels were more than 10 ng/dL (545 pmol/L) higher than the periphery [3].

Cortisol AV:PV ratio is the ratio of cortisol in the AV to the PV; >6.5 was considered evidence for unilateral adrenal production of cortisol [1, 3].

Lateralization ratio: right AV cortisol divided by left AV cortisol; greater than 2.3 considered evidence for unilateral disease.

PV measurements were done with each AV sample. The first listed corresponds to the timing of the right AV sampling, and the second corresponds to the timing of the left AV sampling.

Abbreviations: AV, adrenal vein; PV, peripheral vein.

**Figure 3.**
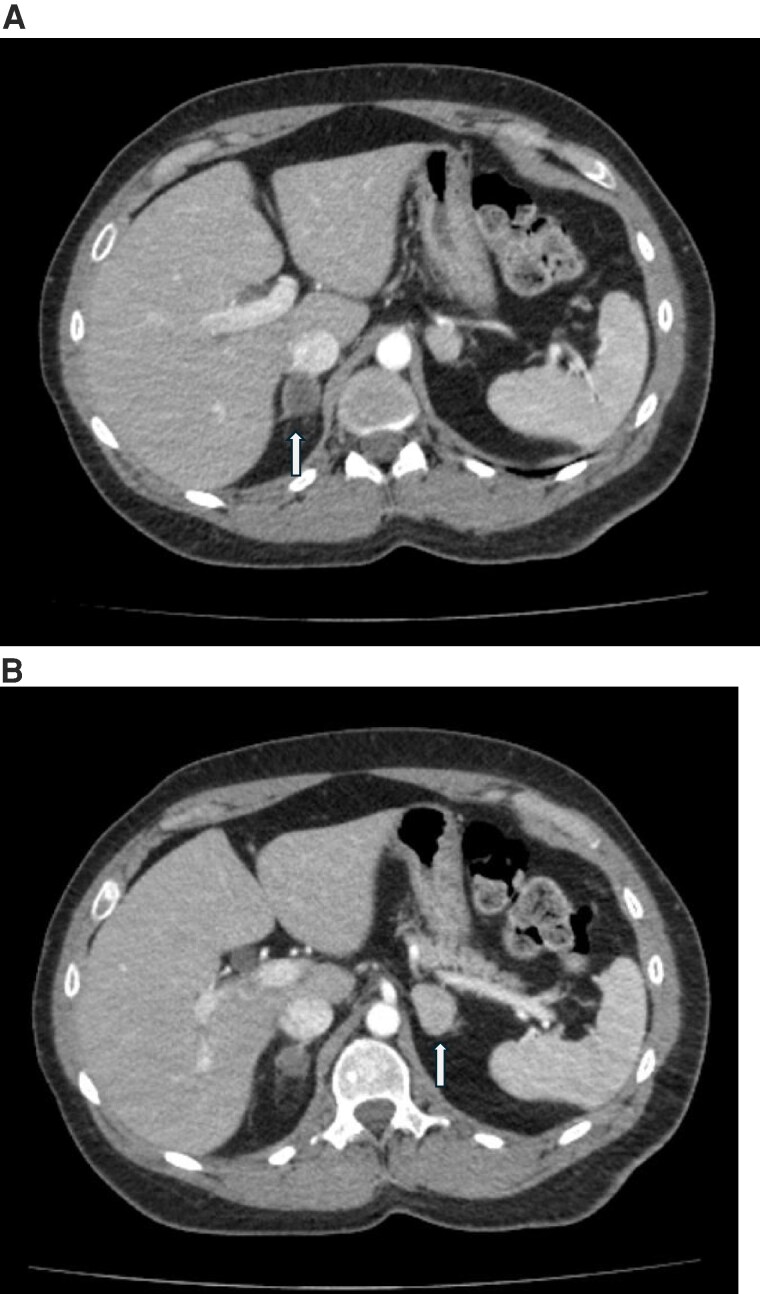
Contrast-enhanced computed tomography of imaging of the patient from December 8, 2020, after adrenal cryoablation (right October 7, 2020) with stable sizes from prior to cryoablation on the right adrenal gland. White arrows show the right and left adrenal glands. (A) Imaging of right adrenal gland; (B) imaging of left adrenal gland.

Follow-up testing over the next 3 months then revealed suppressed ACTH, 2 abnormal midnight salivary cortisol levels, and a 1 mg dexamethasone suppression test cortisol of 7.7 µg/dL; 212.4 nmol/L (reference range less than 1.8 µg/dL; 50 nmol/L) with a dexamethasone level of 267 ng/mL; 6.8 nmol/L (reference range 140-295 ng/dL; 3.6-7.5 nmol/L). Of note, abdominal imaging 2 months after cryoablation of the right adrenal gland showed no change in size of the right or left adrenal nodules (Fig. 3A and 3B) when compared with the images prior to cryoablation (Fig. 2A and 2B).

The patient requested treatment with osilodrostat, a newer medical treatment that is Food and Drug Administration approved for the treatment of pituitary Cushing disease in the United States and endogenous Cushing syndrome in Europe with clinical evidence for efficacy [4]. Osilodrostat decreases cortisol synthesis via inhibition of 11β-hydroxylase, CYP11B1 (11β 1 hydroxylase), less so CYP11B2 (11β 2 hydroxylase), the enzyme responsible for the final step of cortisol biosynthesis in the adrenal gland. Due to its mechanism of action, increases in androgens, deoxycorticosterone, and deoxycortisol can lead to hirsutism and hypertension with hypokalemia. Monitoring for electrocardiogram changes, specifically prolonged QT, and electrolyte disturbances, particularly hypokalemia and hypomagnesemia, is recommended. Dose adjustments may be made no more frequently than every 2 weeks with close monitoring according to the rate of cortisol changes, tolerability, and clinical response.

## Outcome and Follow-up

Osilodrostat was initiated and monitored closely as recommended by the manufacturer. Our patient's 24-hour UFC by LC/MS-MS after dose escalation over 2 months to 6 mg twice daily was unexpectedly elevated at 1043 µg/24 hours; 2878.7 nmol/24 hours (normal range 4-50 µg/24 hours; 11-138 nmol/24 hours). (See Table 2).

**Table 2.** Timeline of Medication Use, Laboratory Values, Symptoms

Date	Medication	ACTH (normal range 6-50 pg/ml; 1.32-11 pmol/L)	Cortisol (normal range 4-22 µg/dL; 110-607 nmol/L)	DHEA-S (normal range 15-205 µg/dL; 408-5578 nmol/L)	24-hour UFC (normal range 4-50 µg/24 hours; 11-138 nmol/24 hours)	Symptoms
2015	None	<5 pg/ml (<1.1 pmol/L)	17.8 µg/dL (491 nmol/L)	N/A	217 µg (599 nmol)	Weight gain, hypertension, hirsutism, acne, irregular menses
Sept. 2015	Unknown mifepristone dose	N/A	N/A	N/A	7.6 µg (21 nmol)	None
Aug. 2021	Mifepristone 900 mg daily	10 pg/ml (2.2 pmol/L)	12.8 µg/dL (353 nmol/L)	48 µg/dL (1306 nmol/L)	11 µg (30 nmol)	Vague symptoms
Sept. 3, 2021	Started osilodrostat 2 mg twice daily	N/A	N/A	N/A	N/A	None
Sept. 17, 2021	Osilodrostat 2 mg twice daily	<5 pg/ml (<1.1 pmol/L)	26.7 µg /dL (737 nmol/L)	102 µg/dL (2776 nmol/L)	372.1 µg (1026.5 nmol)	None
Oct. 4, 2021	Increased osilodrostat 3 mg twice daily	N/A	N/A	N/A	N/A	None
Oct. 18, 2021	Osilodrostat 3 mg twice daily	<5 pg/ml (<1.1 pmol/L)	30.3 µg/dL (836 nmol/L)	138 µg/dL (3755 nmol/L)	912.8 µg (2518 nmol)	Hypertension, hirsutism
Oct. 29, 2021	Increased osilodrostat to 4 mg twice daily	N/A	N/A	N/A	N/A	Hypertension, hirsutism
Nov. 11, 2021	Osilodrostat 4 mg twice daily: increased to 6 mg twice daily	<5 pg/ml (<1.1 pmol/L)	33.7 µg/dL (929 nmol/L)	N/A	1043 µg (2877 nmol)	Hypertension, hirsutism, weight gain
Nov. 29, 2021	Increased osilodrostat to 8 mg twice daily	N/A	N/A	N/A	N/A	Hypertension, hirsutism
Dec. 9, 2021	Osilodrostat 8 mg twice daily, increased to 10 mg twice daily	1.1 pg/ml (0.24 pmol/L)	16.1 µg/dL (444 nmol/L)	N/A	N/A	None
Dec. 28, 2021	Osilodrostat 10 mg twice daily	<5 pg/ml (<1.1 pmol/L)	9.1 µg/dL (251 nmol/L)	10.2 µg/dL (278 nmol/L)	N/A	None
Jan. 26, 2022	Stopped osilodrostat, start hydrocortisone	N/A	2.3 µg/dL (63 nmol/L)	N/A	N/A	Nausea
Feb. 25, 2022	Hydrocortisone 10 mg twice daily	6 pg/ml (1.3 pmol/L)	5.2 µg/dL (143 nmol/L)	N/A	N/A	None
March 3, 2022	Hydrocortisone 10 mg twice daily	42 pg/ml (9.2 pmol/L)	3.5 µg/dL (97 nmol/L)	7 µg/dL (190 nmol/L)	N/A	None
March 23, 2022	Hydrocortisone 10 mg twice daily	25 pg/ml (5.5 pmol/L)	3.9 µg/dL (108 nmol/L)	N/A	N/A	None
May 12, 2022	Hydrocortisone 10 mg twice daily	100 pg/ml (22 pmol/L)	5.6 µg/dL (154 nmol/L)	3 µg/dL 82 nmol/L)	N/A	None
July 11, 2022	Hydrocortisone 10 mg twice daily	79 pg/ml (17.4 pmol/L)	5.5 µg/dL (151 nmol/L)	N/A	4.3 µg (11.9 nmol)	None

Values in parenthesis are International System of Units.

Abbreviations: DHEA-S, dehydroepiandrosterone sulfate; N/A, not available; UFC, urine free cortisol.

During treatment with osilodrostat, there was worsening of Cushing's by laboratory evaluation with increased 24-hour UFC over the initial 8 to 10 weeks of treatment along with worsening symptoms. Hypertension worsened, requiring treatment with spironolactone, and she developed mild hirsutism.

The increase in the urine cortisol prompted dose increases of osilodrostat in a reasonably rapid but closely monitored manner. After she had been on therapy for 4 months, she called the clinic asking for antiemetics. Symptoms of nausea prompted Am cortisol testing that was low at 2.3 µg/dL (63.4 nmol/L). She was then treated with hydrocortisone and followed clinically and biochemically. Unfortunately, her adrenal function did not recover.

**Figure 4.**
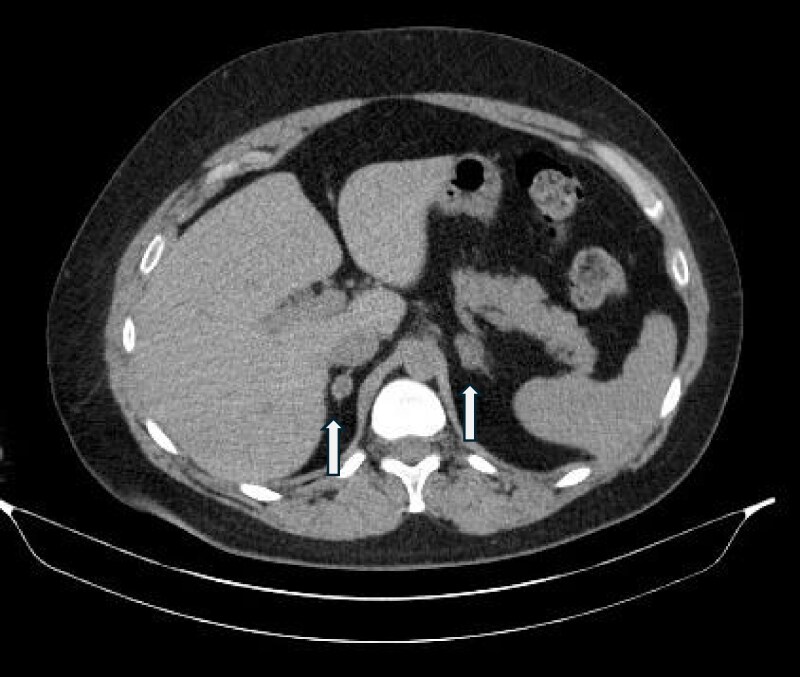
Noncontrast computed tomography imaging of the patient from March 30, 2022, shows that the right adrenal nodule measured 1.1 × 1.1 cm, 20 HU; left adrenal gland measured 2.0 × 1.4 cm (HU not available). White arrows show the right and left adrenal glands. Abbreviation: HU, Hounsfield units.

She has remained primarily adrenally insufficient from December 2021 to the date of this report with elevated ACTH levels and low cortisol and dehydroepiandrosterone sulfate levels with ongoing cortisol replacement requirements. However, her mineralocorticoid function remained intact with normal renin and aldosterone further implicating permanent inhibition of CYP11B1 but not the CYP11B2 enzyme responsible for aldosterone production. A follow-up computed tomography scan of the adrenal glands did show decreased size of the both adrenal glands following treatment with osilodrostat (Fig. 4) when compared with the imaging before (Fig. 2A and 2B) and 2 months after cryoablation (Fig. 3A and 3B). The right adrenal size decreased from 1.7 cm to 1.1 cm and the left from 2.4 cm to 2.0 cm.

The patient did not do well with hydrocortisone due to missing afternoon doses during her workday. Hydrocortisone was discontinued, and she ultimately did well on prednisone 3 mg daily with good control of symptoms. After 3 years, prednisone was tapered and discontinued briefly until she developed syncope and adrenal crisis, prompting resumption of her prednisone.

## Discussion

The response of this patient's cortisol levels to different medical therapies was atypical. Her initial treatment with mifepristone would not have been anticipated to normalize the 24-hour urine cortisol, as its mechanism is to block the glucocorticoid receptor. It also blocks the progesterone receptor. It is possible that the progesterone receptor activity within the adrenal nodule was blocked with mifepristone and that was important in improving the urine cortisol levels. Progesterone receptors have been identified in adrenal tumors of various types [5]. This also could explain why the more specific glucocorticoid receptor blocker relacorilant, which does not have progesterone-blocking activity, did not improve the urine cortisol during the clinical trial.

We cannot explain the rise in her cortisol with osildrostat with our review of the current literature. However, one could suspect that intraadrenal (paracrine) secretion of ACTH was stimulatory [6]. The increase in cortisol following the initiation of osilodrostat was unexpected and prompted dose escalation.

Osilodrostat's mechanism of action is similar to that of metyrapone; however, osilodrostat is a more potent inhibitor of 11β-hydroxylase in vitro and has a longer half-life than metyrapone (4 hours vs 2 hours) [7]. We suspect that the 11β-hydroxylase inhibition action of osilodrostat may have affected the physiologic abnormalities in the hyperplastic adrenal nodule leading to permanent enzymatic blockade of the adrenal corticosteroid biosynthesis pathway. Adrenal insufficiency from osilodrostat has recently been reported in a total of 5 patients [8, 9, 10, 11]. One was treated with osilodrostat for 11 months for persistent Cushing disease before becoming adrenally insufficient [9]. Three other patients treated with osilodrostat for Cushing disease developed prolonged adrenal insufficiency, often treated with a block-and-replace treatment with glucocorticoid replacement and ongoing use of osilodrostat [8]. One report included a patient with adrenal Cushing syndrome treated preoperatively with osilodrostat who developed adrenal insufficiency after 3 weeks of therapy [11].

The utility of the block-and-replace approach to treatment with agents such as osilodrostat has been utilized to allow full adrenal suppression of patients with Cushing syndrome. We note that our patient was keen to avoid the use of any adrenal replacement strategies throughout her care; therefore, this strategy was not employed. In addition, this case was an early experience of the use of osilodrostat, and the block-and-replace treatment was not widely used at that time. However, now it would certainly be a consideration if Cushing syndrome recurs in this patient.

Our patient did have normal renin and aldosterone levels after developing adrenal insufficiency, likely related to the lower affinity of the osilodrostat for the CYP11B2 enzymatic blockade than for the CYP11B1 enzyme.

We recognize the rarity of the development of symptomatic adrenal insufficiency from medical treatment for Cushing syndrome. In this case, the prior treatment of the right adrenal with cryoablation was unsuccessful. Interestingly, the imaging after cryoablation of the right adrenal gland showed no decrease in size. However, computed tomography imaging after treatment with osilodrostat showed a decrease in the size of both adrenal glands. We note that decreased adrenal size associated with osilodrostat treatment has not been reported. Given the timing of the decrease in size that was observed in both adrenal glands and the development of adrenal insufficiency following treatment with osilodrostat, it seems possible that treatment with osilodrostat played a role in the decreased size of both adrenal glands. We wish to bring attention to the possible complication of medication-induced prolonged adrenal insufficiency. Our patient specifically wished to avoid permanent adrenal insufficiency as evidenced by her reluctance to undergo bilateral adrenalectomy surgery at diagnosis and throughout various courses of medical therapy. Urgent management and recognition of symptoms of adrenal insufficiency are mandatory for any patient receiving therapy for cortisol excess.

## Learning Points

- With the availability of many new medical therapies for Cushing disease and syndrome, it is important to recognize rare but urgent side effects.
- Adrenal insufficiency is a potential complication of patients treated with osilodrostat.
- Careful monitoring, symptom recognition, and prompt treatment of this potential complication is necessary.
- Adrenal insufficiency following osilodrostat treatment requires glucocorticoid replacement therapy, and adrenal function should be regularly monitored.

## Data Availability

Data sharing is not applicable to this article as no datasets were generated or analyzed during the current study.
